# Supplementary material for: Cardiovascular disease subtypes, physical disability and workforce participation: A cross-sectional study of 163,562 middle-aged Australians
Source: PLoS One. 2021 Apr 8;16(4):e0249738. doi: 10.1371/journal.pone.0249738 (PMC8031377; doi:10.1371/journal.pone.0249738)
Supplement: S1 File — (DOCX) [file pone.0249738.s001.docx]

# *Bin Sayeed MS, 2021*

**Cardiovascular disease subtypes, physical disability and workforce participation: a cross-sectional study of 163,562 middle-aged Australians**

Muhammad Shahdaat Bin Sayeed*****, Grace Joshy, Ellie Paige, Emily Banks, Rosemary Korda

National Centre for Epidemiology and Population Health, Research School of Population Health, Australian National University, Canberra, ACT, Australia.

***For Correspondence:**

Muhammad Shahdaat Bin Sayeed, ORCID ID: [0000-0003-0027-9614](https://orcid.org/0000-0003-0027-9614)

T: +61 (0)2 612 55618 E-mail: [Muhammad-Shahdaat.Bin-Sayeed@anu.edu.au](mailto:Muhammad-Shahdaat.Bin-Sayeed@anu.edu.au)

National Centre for Epidemiology, Research School of Population Health, Australian National University, Canberra, ACT, Australia

**Short title:** Cardiovascular disease and workforce participation

**Supplementary file tables and figures**

**Supplementary Contents**

| **Tables and Figures** | **Page** |
| --- | --- |

[*Bin Sayeed MS, 2021* 1](#_Toc68025789)

[**S1 Fig**. Flowchart for selection of participants 4](#_Toc68025790)

[**S2 Fig.** Flowchart for selection of participants included in the analysis to estimate the effect size for the association of CVD and workforce participation status 6](#_Toc68025791)

[**S1 Table.** The study participants number based on workforce participation-based classification 7](#_Toc68025792)

[**S3 Fig.** Flowchart for selection of participants included in the analysis to estimate the effect size for the association of CVD and paid work hour per week 8](#_Toc68025793)

[**S4 Fig.** Flowchart for selection of participants included in the analysis to estimate the effect size for the association of CVD and retirement due to ill health 9](#_Toc68025794)

[**S2 Table.** The number of participants according to participating in the workforce and retirement 10](#_Toc68025795)

[**S3 Table.** The number of participants according to CVD and retirement status 11](#_Toc68025796)

[S4 Table. Workforce participation, retirement and retirement due to ill health 12](#_Toc68025797)

[S5 Table. Codes for CVD selection from hospitalization records 13](#_Toc68025798)

[S6 Table. The physical functional limitations calculation and categorisation 15](#_Toc68025799)

[**S7 Table.** Non-participation in the workforce: prevalence and adjusted prevalence ratios according to CVD status where CVD was defined based on hospitalisation records and self-reported survey 16](#_Toc68025800)

[**S8 Table.** Non-participation in the workforce: prevalence and adjusted prevalence ratios according to CVD status excluding those with multiple CVD subtypes 17](#_Toc68025801)

[**S9 Table.** Non-participation in the workforce: prevalence and adjusted prevalence ratios according to different CVD-subtype in combination defined based on hospitalisation records 18](#_Toc68025802)

[**S5 Fig.** Paid hours of work per week: means and mean differences according to CVD status among those in paid work 19](#_Toc68025803)

[**S10 Table.** Paid hours of work per week: means and mean differences according to CVD status where CVD was defined based on hospitalisation records and self-reported survey 20](#_Toc68025804)

[**S11 Table.** Paid hours of work per week: means and mean differences according to CVD status among those in paid work excluding those with multiple CVD subtypes 21](#_Toc68025805)

[**S12 Table.** Paid hours of work per week*: means and mean differences according to different CVD-subtype in combination defined based on hospitalisation records 22](#_Toc68025806)

[**S6 Fig.** Retirement: prevalence and adjusted prevalence ratios according to CVD subtype 23](#_Toc68025807)

[**S13 Table.** Retirement: prevalence and adjusted prevalence ratios according to CVD status where CVD was defined based on hospitalisation records and self-reported survey 24](#_Toc68025808)

[**S14 Table.** Retirement: prevalence and adjusted prevalence ratios according to CVD status excluding those with multiple CVD subtypes 25](#_Toc68025809)

[**S15 Table.** Retirement*: prevalence and adjusted prevalence ratios according to different CVD-subtype in combination defined based on hospitalisation records 26](#_Toc68025810)

[**S7 Fig.** Retirement due to ill health: prevalence and adjusted prevalence ratios according to CVD subtype among those who have retired and who had not been in paid workforce 27](#_Toc68025811)

[**S16 Table.** Retirement due to ill health: prevalence and adjusted prevalence ratios according to CVD status where CVD was defined based on hospitalisation records and self-reported survey 28](#_Toc68025812)

[**S17 Table.** Retirement due to ill health: prevalence and adjusted prevalence ratios according to CVD status excluding those with multiple CVD subtypes 29](#_Toc68025813)

[**S18 Table.** Retirement due to ill health*: prevalence and adjusted prevalence ratios according to different CVD-subtype in combination defined based on hospitalisation records 30](#_Toc68025814)

[**S8 Fig.** Relation of sociodemographic factors to non-participation in the workforce among those with and without CVD 31](#_Toc68025815)

## **S1 Fig**. Flowchart for selection of participants

**Steps of selecting the number of participants in different exposure-outcome associations**

The depth of the analysis as presented in this paper required to select whole or portions of the participants which were broadly mentioned in four stages. The study participants selected in stage 1 were used for presenting the characteristics of the study participants. The number of participants used in the final exposure-outcome association analysis varied and it depended on the corresponding outcomes. The stages and the number of participants in respective analysis are as follows:

- **Stage 1:** Selecting eligible study participants who were aged less than 65 years old. There were 163562 participants finally selected who were used for describing the characteristics of the study participants (***S1 Fig***).
- **Stage 2:** Getting the study participants who had non-missing workforce participation status to investigate the association of CVD and workforce participation status by considering CVD subtypes, population sub-groups and physical functioning limitations. There were 131 participants with missing workforce participation status. They were excluded and the final analysis that investigated the association of CVD and workforce participation status included 163431 participants (***S2 Fig***).
- **Stage 3:** Divide the study participants into two groups and continue further investigations in those groups separately. We have divided into two groups based on workforce participation status (**S1 Table**) and investigated paid work hour per week in those who had been working (and had valid paid work hour per week) and retired due to ill health in those who had not been working (and had valid record of retirement).

*Group 1:* Getting the participants who had been working and had valid weekly paid work hour. Then the associations of CVD and paid work hours per week among the selected participants were investigated. The final analysis that studied the association of CVD and paid work hours per week included 114064 participants (***S3 Fig***).

*Group 2:* Getting the participants who had not been working and had valid retirement due to ill-health record as outcome. Then the association of CVD and retirement due to ill health among the selected participants who had not working in any form were studied. The final analysis that investigated the association of CVD and paid work hour per week included 114064 participants (***S4 Fig***).

- **Stage 4:** The final analysis was to investigate the association of CVD and retirement. There were some confliction arised after cross-tabulation of workforce participation status and retirement. For example, 11961 participants were grouped as both ‘Not in paid workforce’ and ‘Not retired’ category, and 13645 participants were grouped as both ‘In paid workforce’ and ‘retired’ (**S2 Table)**. However, we have not considered this apparent conflicting issue while investigating the association of CVD and retirement. Since there was no missing data for retirement, the final analysis that investigated the association of CVD and retirement included 163562 participants (**S3 Table**).

| Study participants selection steps | CVD group | No CVD group | Total | Comments |
| --- | --- | --- | --- | --- |
| Eligible participants from the 45 and Up Study who were <65 years old | N= 19161 | N= 144401 | N= 163562 |  |
|  |  |  |  |  |
| Participants having missing workforce participation status | N= 24 | N= 107 | N= 131 | Excluded |
|  |  |  |  |  |
| Participants having valid workforce participation status | N= 19137 | N= 144294 | N= 163431 | Finally included |
|  |  |  |  |  |

## **S2 Fig.** Flowchart for selection of participants included in the analysis to estimate the effect size for the association of CVD and workforce participation status

## **S1 Table.** The study participants number based on workforce participation-based classification

| Groups | Workforce participation status | Number of participants |
| --- | --- | --- |
| Group 1 | In workforce | N= 121816 |
| Group 2 | Not in workforce | N= 41615 |
| Total participants |  | N= 163431 |

| Study participants | CVD group | No CVD group | Total | Comments |
| --- | --- | --- | --- | --- |
| Eligible participants from the 45 and Up Study who were <65 years old and working | N=11480 | N=110336 | N= 121816 |  |
| Participants having missed paid work hours per week | N=972 | N=6736 | N= 7708 | Excluded |
|  |  |  |  |  |
| Participants having more than or equal to 100 hours of work per week. We defined these outliers and thus considered missing | N=2 | N=42 | N= 44 | Excluded |
|  |  |  |  |  |
| Participants having valid paid work hours per week | N= 10506 | N= 103558 | N= 114064 | Finally included |

## **S3 Fig.** Flowchart for selection of participants included in the analysis to estimate the effect size for the association of CVD and paid work hour per week

| Study participants | CVD group | No CVD group | Total | Comment |
| --- | --- | --- | --- | --- |
| Eligible participants from the 45 and Up Study who were <65 years old and not working | N= 7657 | N= 33958 | N= 41615 |  |
|  |  |  |  |  |
| Participants who had been grouped as ‘not retired’. This is because retired and workforce participation status were defined based on different questions sets. Since the same person being ‘not in work’ and ‘not retired’ is conflicting, we have considered these as invalid/missing. | N=1687 | N=10274 | N= 11961 | Excluded |
|  |  |  |  |  |
|  |  |  |  |  |
| Participants having valid record on retirement due to ill health or not | N= 5970 | N= 23684 | N= 29654 | Finally included |

## **S4 Fig.** Flowchart for selection of participants included in the analysis to estimate the effect size for the association of CVD and retirement due to ill health

## **S2 Table.** The number of participants according to participating in the workforce and retirement

|  | Workforce participation status | Retirement status | | Total |
| --- | --- | --- | --- | --- |
|  |  | Not retired | Retired |  |
|  | Not in paid workforce | N= 11961 | N= 29654 | N= 41615 |
|  | In Paid workforce | N= 108171 | N= 13645 | N= 121816 |
|  | Missing | N= 33 | N= 98 | N= 131 |
| Total |  | N= 120165 | N= 43397 | N= 163562 |

## **S3 Table.** The number of participants according to CVD and retirement status

|  | CVD group | No CVD group | Total |
| --- | --- | --- | --- |
| Not Retired | N= 11186 | N= 108979 | N= 120165 |
| Retired | N= 7975 | N= 35422 | N= 43397 |
| Total | N= 19161 | N= 144401 | N= 163562 |

## S4 Table. Workforce participation, retirement and retirement due to ill health

| The main outcome of interest was non-participation in paid work (yes/no). We also reported on paid work hours/week among those in paid work, retirement of all working age participants (yes/no), and retirement due to ill-health (yes/no) among the retirees who were not in the workforce. The outcomes as mentioned in this investigation are based on the 3 questions in the 45 and Up Study [1] as follows:  **Question 47: What is your current work status?** (you can cross more than one box)   \| - In full time paid work - In part time paid work - Completely retired/pensioner - Partially retired - Disabled/sick - Other \| - Self-employed - Doing unpaid work - Studying - Looking after home/family - Unemployed \| \| --- \| --- \|   **Question 48: If you are partially or completely retired, why did you retire?**   \| - Reached usual retirement age - To care for family members/friend - Made redundant - Other \| - Lifestyle reasons - Ill health - Made redundant - Could not find a job \| \| --- \| --- \|   **Question 49: About how many HOURS each week do you usually spend doing the following:**  hours per week   \|  \|  \| paid work \| \| --- \| --- \| --- \|  - **Paid hours of work per week**   This is a count variable consisting of zero or non-zero positive values obtained from question number 49 in the 45 and Up study. Those having more than value more than 100 or negative values as recorded in the survey were considered as invalid and hence considered missing in the survey.   - **Workforce participation**   This is a binary outcome obtained from question number 47 and 49 in the 45 and Up study generating two options: yes versus no. Those indicating valid paid hours (≥ 0 and <100) or work status (current work status as at least one of “In full time paid work”, “In part time paid work”, “Self-employed”, “Partially retired”) were classified as participating in the workforce, and others (“Doing unpaid work”, “Completely retired/pensioner”, “Studying”, “Looking after home/family”, “Disabled/sick”, “Unemployed”, “Other”) were classified as not participating in the workforce. The steps of defining workforce participation were as follows:   1. Participants were considered to be in paid work if:    1. Number of hours of paid work hours is valid (0 to <100) OR    2. Reported being in full time paid work, in part time paid work, self-employed or partially retired. 2. Participants were considered to be not in paid work if:    1. Number of hours of paid work hours per week is zero (0) or missing (but not invalid) AND    2. Not reported being in fulltime paid work, part time paid work, self-employed or partially retired 3. People who are not paid for work automatically received zero (0) for paid work hour per week 4. For those who are in “paid” category and entered 0 as paid work hour per week, their paid work status was accepted, and weekly paid work hours were invalidated 5. For those who are in “not paid” category and entered valid paid work hours per week, their paid work status was changed, and weekly paid work hours were accepted when the weekly paid work hours were larger than 0.  - **Retirement**   This is a binary outcome obtained from question number 48 in the 45 and Up study generating two options: yes versus no. Participants with valid records for any of the options (“Reached usual retirement age”, “Lifestyle reasons”, “To care for family members/friend”, “Ill health”, “Made redundant”, “Made redundant”, “Could not find a job”, “Other”) were defined as ‘retired’ and those without any of these options were defined as ‘not retired’.   - **Retirement due to ill health**   This is a binary outcome derived from question number 47, 48 and 49 in the 45 and Up study generating two options: yes versus no. The binary definition of retirement and workforce participation status resulted in some participants who had been categorised as both ‘retirees’ and ‘participating in paid workforce’. Hence, to indicate those who had fully retired and not participating in the paid workforce, the participants who had been defined as participating in the workforce among the retirees were excluded. Then among the fully retirees who had not been working, reasons for retirement were classified as binary outcome: ‘retirement due to ill health’ and ‘retirement due to other reasons’ (“Reached usual retirement age”, “Lifestyle reasons”, “To care for family member/friend”, “Made redundant”, “Could not find a job”, “Other”).  *Reference*  1. Banks, E., et al., *Cohort profile: the 45 and up study.* Int J Epidemiol, 2008. **37**(5): p. 941-7. |
| --- | --- | --- | --- | --- | --- | --- | --- |

## S5 Table. Codes for CVD selection from hospitalization records

| 1. **CVD includes those with either ICD-10-AM codes or coronary procedures codes as follows** 2. *ICD-10-AM codes*   I11-I13  120-I25  I26-I28  I34-36,  I42  I44  I46-I51  I61-I67  I69  I70-I77  I80  G45  G46   1. *Coronary procedure codes*   *Percutaneous coronary interventions;  35304-00, 35305-00, 35304-01, 35305-01,35310-00, 35310-01, 35310-02,35310-03, 35310-04, 35310-05, 38300-00, 38303-00, 38306-00, 38306-01, 38306-02, 38306-03, 38306-05  *Coronary artery bypass grafting;  38497-00 to 38497-07  38500-00 to 38500-04  38503-00, 38503-01  90201-01 to 90201-03,  *Heart transplant:  90205-00, 90205-01  *Cardiac defibrillator implants:  38524-00, 38521-01, 38521-02, 38521-03, 38393-00  *Valve replacement, repair or reconstruction:  38456-10, 38483-00, 38270-01, 38480-00, 38481-00, 38488-00, 38488-01, 38489-00, 38489-01,38456-15, 38653-04, 38475-02, 38477-02,38487-00, 38485-01, 38270-02, 38480-01, 38481-01, 38475-00, 38477-00,38488-02, 38488-03, 38489-02,38485-00, 38456-16, 38653-05, 38456-11, 38480-02, 38481-02, 38475-01, 38477-01,38488-04, 38488-05,38489-03, 38456-17, 38653-06, 38456-01, 38270-03, 38488-06, 38488-07, 38489-04, 38489-05, 38456-18, 38653-07  *pacemaker insertion:  38281-00, 38281-01, 38281-02, 38281-03, 38281-04, 38281-05, 38281-06, 38281-07,38281-08, 38281-09, 38281-10, 38281-11, 38281-12, 38281-13, 38353-00  *Carotid endarterectomy:  33500-00   1. **CVD Subtypes codes** 2. Ischaemic heart disease based on ICD-10-AM codes:   I20-I25   1. Cerebrovascular disease based on ICD-10-AM codes:   I61-I67, I69   1. Myocardial infarction based on ICD-10-AM codes:   I22-I23   1. Heart Failure based on ICD-10-AM codes:   I50   1. Peripheral Arterial Disease based on ICD-10-AM codes:   I70-I74, I77   1. **Other CVD**   Includes those who had self-reported CVD from the 45 and Up study survey or had any ICD-10-AM codes or coronary procedures codes as mentioned in **A** as above except those as mentioned in **B** as above. |
| --- |

## S6 Table. The physical functional limitations calculation and categorisation

| Physical functioning was measured using the Medical Outcomes Score‐Physical Functioning (MOS‐PF)[1], which is equivalent to items from the physical functioning scale (PF‐10) of the SF‐36 health survey[2]. The PF‐10 has been validated as a measure of physical functioning across a wide range of patient groups varying by age, sex, and comorbidities [3]. It consists of 10 questionnaire items and asks the study participants to choose one of the three choices ‘Yes, limited a lot’, ‘Yes, a little’ or ‘No, not limited at all’ in response to the question:  “Does your health now limit you in any of the following activities?” with a list of 10 activities as follows:   1. VIGOROUS activities (e.g running, strenuous sports) 2. MODERATE activities (e.g pushing a vacuum cleaner, playing golf) 3. Lifting or carrying shopping 4. Climbing several flights of stairs 5. Climbing one flights of stairs 6. Walking one kilometre 7. Walking half a kilometre 8. Walking 100 metres 9. Bending, kneeling or stooping 10. Bathing or dressing yourself   For each item, participants answer “yes, limited a lot,” “yes, limited a little,” or “no, not limited at all,” had score of 0, 50, or 100 respectively. An overall physical functioning score was calculated from the average of scores from all 10 items. Therefore, the PFL scores ranged from 0 to 100, where higher scores represented fewer limitations, and were grouped into four categories: no limitation (score of 100); minor limitation (score 90–<100); moderate limitation (60–<90); and severe limitation (score 0–<60). Such cut-off values were chosen in refence to previously published research [4-6]  **Reference**  1. Stewart AL, Ware JE. Measuring functioning and well-being: the medical outcomes study approach: duke university Press; 1992.  2. Ware JE Jr, Sherbourne CD. The MOS 36-item short-form health survey (SF-36). I. Conceptual framework and item selection. Med Care. 1992 Jun;30(6):473-83. PMID: 1593914  3. Haley SM, McHorney CA, Ware JE Jr. Evaluation of the MOS SF-36 physical functioning scale (PF-10): I. Unidimensionality and reproducibility of the Rasch item scale. J Clin Epidemiol. 1994 Jun;47(6):671-84. doi: 10.1016/0895-4356(94)90215-1. PMID: 7722580  4. Gubhaju L, Banks E, MacNiven R, McNamara BJ, Joshy G, Bauman A, Eades SJ. Physical Functional Limitations among Aboriginal and Non-Aboriginal Older Adults: Associations with Socio-Demographic Factors and Health. PLoS One. 2015 Sep 30;10(9):e0139364. doi: 10.1371/journal.pone.0139364. PMID: 26422239  5. Zhang Y, Joshy G, Glass K, Banks E. Physical functional limitations and psychological distress in people with and without colorectal cancer: findings from a large Australian study. J Cancer Surviv. 2020 Dec;14(6):894-905. doi: 10.1007/s11764-020-00901-y. Epub 2020 Jul 2. PMID: 32613443  6. Gardiner PA, Reid N, Gebel K, Ding D. Sitting Time and Physical Function in Australian Retirees: An Analysis of Bidirectional Relationships. J Gerontol A Biol Sci Med Sci. 2018 Nov 10;73(12):1675-1681. doi: 10.1093/gerona/gly008. PMID: 29408975 |
| --- |

**Sensitivity analysis-I:**

## **S7 Table.** Non-participation in the workforce: prevalence and adjusted prevalence ratios according to CVD status where CVD was defined based on hospitalisation records and self-reported survey

|  | **Not in workforce**  **% [n/N]** | **Prevalence ratio (95% CI)** | |
| --- | --- | --- | --- |
|  |  | **Model^1^** | **Model^2^** |
| Hospital recorded CVD (regardless of self-reported CVD) ^a^ | 43.0 (3720/8659) | 1.55 (1.52-1.59) | 1.46 (1.42-1.50) |
| Self-reported CVD (regardless of hospitalisation recorded CVD) ^b^ | 40.1 (6355/15852) | 1.43 (1.40-1.46) | 1.36 (1.34-1.39) |
|  |  |  |  |
| Hospital recorded CVD only (excluding those with self-reported CVD) ^c^ | 39.6 (1302/3285) | 1.43 (1.37-1.49) | 1.34 (1.29-1.40) |
| Self-reported CVD only (excluding those with hospitalisation recorded CVD) ^c^ | 37.6 (3937/10478) | 1.33 (1.30-1.36) | 1.28 (1.25-1.31) |
|  |  |  |  |
| Both self-reported and hospitalisation recorded CVD ^c^ | 45.0 (2418/5374) | 1.63 (1.59-1.68) | 1.53 (1.48-1.58) |
|  |  |  |  |
| Main analysis CVD ^c^ | 40.0 (7657/19137) | 1.43 (1.40-1.46) | 1.36 (1.33-1.39) |
| No self-reported or hospital recorded CVD ^c^ (Ref) | 23.5 (33958/144294) | 1 | 1 |

^1^Adjusted for age and sex. ^2^Further adjusted for remoteness of residence and education.

^a^Based on hospital admission only, ^b^ Based on self-report only, ^c^ Based on both self-report and hospital admission

**Sensitivity analysis-II**

## **S8 Table.** Non-participation in the workforce: prevalence and adjusted prevalence ratios according to CVD status excluding those with multiple CVD subtypes

|  | **Not in workforce**  **% [n/N]** | **Prevalence ratio (95% CI)** | |
| --- | --- | --- | --- |
| **Total n/N** | 25.5 (41615/163431) | **Model^1^** | **Model^2^** |
| CVD ^a^ | 40.0 (7657/19137) | 1.43 (1.40-1.46) | 1.36 (1.33-1.39) |
| *Ischaemic heart disease only b* | 41.8 (1260/3017) | 1.49 (1.42-1.55) | 1.39 (1.34-1.45) |
| *Myocardial infarction only b* | 36.1 (382/1059) | 1.45 (1.35-1.57) | 1.34 (1.24-1.44) |
| *Cerebrovascular disease only b* | 55.0 (280/509) | 1.99 (1.83-2.16) | 1.85 (1.70-2.01) |
| *Peripheral arterial diseases only b* | 52.1 (214/411) | 1.81 (1.65-1.98) | 1.66 (1.52-1.82) |
| *Heart failure only b* | 49.7 (91/183) | 1.77 (1.52-2.05) | 1.51 (1.29-1.75) |
| No CVD (reference) | 23.5 (33958/144294) | 1 | 1 |

^1^Adjusted for age and sex. ^2^Further adjusted for remoteness of residence and education.

^a^ Based on self-report and hospital records ^b^Based on hospital records only and participants with CVD subtypes other than the named particular type of CVD subtype were excluded. Effect sizes were estimated using ‘no CVD’ as the reference group.

**Sensitivity analysis-III:**

## **S9 Table.** Non-participation in the workforce: prevalence and adjusted prevalence ratios according to different CVD-subtype in combination defined based on hospitalisation records

|  | **Not in workforce % [n/N]** | **Prevalence ratio (95% CI)** | |
| --- | --- | --- | --- |
|  |  | **Model^1^** | **Model^2^** |
| IHD and Cerebrovascular disease combined ^a^ | 65.7 (94/143) | 2.33(2.05-2.64) | 2.09 (1.82-2.40) |
| IHD and HF combined ^a^ | 69.2 (164/237) | 2.35 (2.14-2.57) | 2.07(1.89-2.27) |
| IHD, HF and PAD combined ^a^ | 81.3 (26/32) | 2.55(2.14-3.05) | 2.12 (1.77-2.55) |
| Stroke and HF combined ^a^ | 84.4 (27/32) | 2.95 (2.48-3.50) | 2.62 (2.18-3.16) |
| IHD, Cerebrovascular disease and PAD combined ^a^ | 66.7 (16/24) | 2.10 (1.56-2.82) | 2.10 (1.50-2.94) |
|  |  |  |  |
| Main analysis CVD ^b^ | 40.0 (7657/19137) | 1.43 (1.40-1.46) | 1.36 (1.33-1.39) |
| No self-reported or hospital recorded CVD ^b^ (Ref) | 23.5 (33958/144294) | 1 | 1 |

^*^Combination of different subtypes were reported where sufficient numbers were available.

^1^Adjusted for age and sex. ^2^Further adjusted for remoteness of residence and education.

^a^Hospital admission only, ^b^ both self-report and hospital admission, IHD= Ischaemic heart disease, HF= Heart failure, PAD= Peripheral arterial disease

**(Supplement analysis)**

## **S5 Fig.** Paid hours of work per week: means and mean differences according to CVD status among those in paid work

|  | **Total N** | **Mean (95% CI)** | **Difference in Mean (95% CI)** | |  |
| --- | --- | --- | --- | --- | --- |
|  | 114064 |  | **Model^1^** | **Model^2^** |  |
| CVD ^a^ | 10506 | 34.9 (34.6, 35.2) | -0.95 (-1.13, -0.77) | -0.92 (-1.02, -0.82) |  |
| *Ischaemic heart disease ^b^* | 2406 | 36.7 (36.0, 37.3) | -0.69 (-1.14, -0.23) | -0.62 (-0.95, -0.28) |  |
| *Myocardial infarction ^b^* | 679 | 36.9 (35.8, 38.1) | -1.50 (-2.38, -0.58) | -1.23 (-1.98, -0.46) |  |
| *Cerebrovascular disease ^b^* | 263 | 32.9 (31.0, 34.9) | -3.39 (-5.10, -1.57) | -3.40 (-4.98, -1.72) |  |
| *Peripheral arterial diseases ^b^* | 265 | 34.7 (32.7, 36.6) | -1.24 (-2.88, 0.49) | -1.16 (-2.65, 0.42) |  |
| *Heart failure ^b^* | 156 | 34.7 (32.0, 37.3) | -1.98 (-4.21, 0.42) | -1.45 (-3.53, 0.81) |  |
| *Other CVD* ^a^ | 7612 | 34.5 (34.1, 34.8) | -0.95 (-1.18, -0.72) | -0.94 (-1.08, -0.80) |  |
| No CVD (reference) | 103558 | 35.9 (35.8, 36.0) | 0 | 0 |  |
|  |  |  |  |  |  |

^1^Adjusted for age and sex. ^2^Adjusted for age, sex, remoteness of residence and education.

^a^ Based on self-report and hospital records ^b^Based on hospital records only and regardless of presence of other CVD subtypes.

Effect sizes were estimated using ‘no CVD’ as the reference group.

**(Supplement analysis: Sensitivity analysis-I)**

## **S10 Table.** Paid hours of work per week: means and mean differences according to CVD status where CVD was defined based on hospitalisation records and self-reported survey

|  | **Total N** | **Mean (95% CI)** | **Difference in Mean (95% CI)** | |
| --- | --- | --- | --- | --- |
|  |  |  |  |  |
|  |  |  | **Model^1^** | **Model^2^** |
| Hospital recorded CVD (regardless of self-reported CVD) ^a^ | 4522 | 35.8 (35.3, 36.2) | -0.95 (-1.26, -0.64) | -0.90 (-1.11, -0.69) |
| Self-reported CVD (regardless of hospitalisation recorded CVD) ^b^ | 8686 | 34.8 (34.5, 35.1) | -1.01 (-1.22, -0.80) | -0.98 (-1.11, -0.86) |
|  |  |  |  |  |
| Hospital recorded CVD only (excluding those with self-reported CVD) ^c^ | 1820 | 35.6 (34.9, 36.2) | -0.68 (-1.21, -0.14) | -0.64 (-1.05, -0.22) |
| Self-reported CVD only (excluding those with hospitalisation recorded CVD) ^c^ | 5984 | 34.3 (33.9, 34.7) | -0.96 (-1.23, -0.68) | -0.95 (-1.12, -0.77) |
|  |  |  |  |  |
| Both self-reported and hospitalisation recorded CVD ^c^ | 2702 | 35.9 (35.3, 36.5) | -1.14 (-1.57, -0.70) | -1.08 (-1.39, -0.76) |
|  |  |  |  |  |
| Main analysis CVD ^c^ | 10506 | 34.9 (34.6, 35.2) | -0.95 (-1.13, -0.77) | -0.92 (-1.02, -0.82) |
| No self-reported or hospital recorded CVD ^c^ (Ref) | 103558 | 35.9 (35.8, 36.0) | 0 | 0 |

^1^Adjusted for age and sex. ^2^Further adjusted for remoteness of residence and education. ^a^Based on hospital admission only, ^b^ Based on self-report only, ^c^ Based on both self-report and hospital admission

**(Supplement analysis: Sensitivity analysis-II)**

## **S11 Table.** Paid hours of work per week: means and mean differences according to CVD status among those in paid work excluding those with multiple CVD subtypes

|  | **Total N** | **Mean (95% CI)** | **Difference in Mean (95% CI)** | |
| --- | --- | --- | --- | --- |
| **Total N** | 114064 |  | **Model^1^** | **Model^2^** |
| CVD ^a^ | 10506 | 34.9 (34.6, 35.2) | -0.95 (-1.13, -0.77) | -0.92 (-1.02, -0.82) |
| *Ischaemic heart disease only b* | 1613 | 36.7 (35.9, 37.4) | -0.26 (-0.83, 0.33) | -0.29 (-0.74, 0.17) |
| *Myocardial infarction only b* | 625 | 37.0 (35.8, 38.2) | -1.53 (-2.46, -0.57) | -1.23 (-2.02, -0.41) |
| *Cerebrovascular disease only b* | 210 | 32.7 (30.6, 34.9) | -3.40 (-5.28, -1.39) | -3.34 (-5.08, -1.47) |
| *Peripheral arterial diseases only b* | 179 | 33.9 (31.4, 36.4) | -1.76 (-3.86, 0.50) | -1.81 (-3.75, 0.30) |
| *Heart failure only b* | 82 | 35.4 (31.5, 39.3) | -1.06 (-4.27, 2.52) | -0.62 (-3.72, 2.85) |
| No CVD (reference) | 103558 | 35.9 (35.8, 36.0) | 0 | 0 |

Model^1^= Adjusted for age and sex, Model^2^ = Further adjusted for remoteness of residence and education attainment. ^b^Based on hospital records only and participants with CVD subtypes other than the named particular type of CVD subtype were excluded, Effect sizes were estimated using ‘no CVD’ as the reference group.

**(Supplement analysis: Sensitivity analysis-III)**

## **S12 Table.** Paid hours of work per week*: means and mean differences according to different CVD-subtype in combination defined based on hospitalisation records

|  | **Total N** | **Mean (95% CI)** | **Difference in Mean (95% CI)** | |
| --- | --- | --- | --- | --- |
|  |  |  | **Model^1^** | **Model^2^** |
| IHD and Cerebrovascular disease combined ^a^ | 43 | 34.1 (28.3, 40.0) | -2.88 (-7.58, 2.66) | -3.37 (-7.99, 2.14) |
| IHD and HF combined ^a^ | 64 | 33.5 (29.5, 37.5) | -3.24 (-6.57, 0.50) | -2.66 (-5.75, 0.82) |
|  |  |  |  |  |
| Main analysis CVD ^c^ | 10506 | 34.9 (34.6, 35.2) | -0.95 (-1.13, -0.77) | -0.92 (-1.02, -0.82) |
| No self-reported or hospital recorded CVD ^c^ (Ref) | 103558 | 35.9 (35.8, 36.0) | 0 | 0 |

^*^Combination of different subtypes were reported where sufficient numbers were available.

^1^Adjusted for age and sex. ^2^Further adjusted for remoteness of residence and education.

^a^Hospital admission only, ^b^ both self-report and hospital admission, IHD= Ischaemic heart disease, HF= Heart failure

**(Supplement analysis)**

## **S6 Fig.** Retirement: prevalence and adjusted prevalence ratios according to CVD subtype

|  | **Retired % (n/N)** | **Prevalence ratio (95% CI)** | |  |
| --- | --- | --- | --- | --- |
| **Total n/N** | 26.5 (43397/163562) | **Model^1^** | **Model^2^** |  |
| CVD ^a^ | 41.6 (7975/19161) | 1.28 (1.25-1.30) | 1.25 (1.23-1.28) |  |
| *Ischaemic heart disease ^b^* | 45.2 (2084/4609) | 1.31 (1.27-1.35) | 1.28 (1.24-1.32) |  |
| *Myocardial infarction ^b^* | 42.1 (518/1231) | 1.31 (1.23-1.39) | 1.27 (1.20-1.35) |  |
| *Cerebrovascular disease ^b^* | 55.9 (391/700) | 1.66 (1.55-1.77) | 1.61 (1.51-1.72) |  |
| *Peripheral arterial diseases ^b^* | 55.2 (378/685) | 1.56 (1.46-1.66) | 1.50 (1.41-1.61) |  |
| *Heart failure ^b^* | 56.8 (252/444) | 1.63 (1.50-1.77) | 1.54 (1.42-1.67) |  |
| *Other CVD* ^a^ | 39.5 (5292/13387) | 1.24 (1.21-1.26) | 1.22 (1.20-1.25) |  |
| No CVD (reference) | 24.5 (35422/144401) | 1 | 1 |  |
|  |  |  |  |  |
|  |  |  |  | Prevalence ratio (95% CI) on log scale |

Model^1^= Adjusted for age and sex, Model^2^ = Further adjusted for remoteness of residence and education attainment. ^a^Based on both self-reported survey and hospital records, ^b^Based on hospital records only and regardless of other CVD diagnosis. Effect sizes were estimated using ‘no CVD’ as the reference group.

**(Supplement analysis: Sensitivity analysis-I)**

## **S13 Table.** Retirement: prevalence and adjusted prevalence ratios according to CVD status where CVD was defined based on hospitalisation records and self-reported survey

|  | **Retired**  **% [n/N]** | **Prevalence ratio (95% CI)** | |
| --- | --- | --- | --- |
|  |  | **Model^1^** | **Model^2^** |
| Hospital recorded CVD (regardless of self-reported CVD) ^a^ | 44.3 (3840/8670) | 1.32 (1.29-1.35) | 1.30 (1.27-1.33) |
| Self-reported CVD (regardless of hospitalisation recorded CVD) ^b^ | 42.2 (6691/15873) | 1.29 (1.27-1.31) | 1.27 (1.24-1.29) |
|  |  |  |  |
| Hospital recorded CVD only (excluding those with self-reported CVD) ^c^ | 39.1 (1284/3288) | 1.21 (1.16-1.26) | 1.18 (1.14-1.23) |
| Self-reported CVD only (excluding those with hospitalisation recorded CVD) ^c^ | 39.4 (4135/10491) | 1.24 (1.21-1.27) | 1.22 (1.19-1.25) |
|  |  |  |  |
| Both self-reported and hospitalisation recorded CVD ^c^ | 47.5 (2556/5382) | 1.39 (1.35-1.43) | 1.36 (1.32-1.40) |
|  |  |  |  |
| Main analysis CVD ^c^ | 41.6 (7975/19161) | 1.28 (1.25-1.30) | 1.25 (1.23-1.28) |
| No self-reported or hospital recorded CVD ^c^ (Ref) | 24.5 (35422/144401) | 1 | 1 |

^1^Adjusted for age and sex. ^2^Further adjusted for remoteness of residence and education.

^a^Based on hospital admission only, ^b^ Based on self-report only, ^c^ Based on both self-report and hospital admission

**(Supplement analysis: Sensitivity analysis-II)**

## **S14 Table.** Retirement: prevalence and adjusted prevalence ratios according to CVD status excluding those with multiple CVD subtypes

|  | **Retired**  **% [n/N]** | **Prevalence ratio (95% CI)** | |
| --- | --- | --- | --- |
| **Total n/N** | 26.5 (43397/163562) | **Model^1^** | **Model^2^** |
| CVD ^a^ | 41.6 (7975/19161) | 1.28 (1.25-1.30) | 1.25 (1.23-1.28) |
| *Ischaemic heart disease only b* | 44.1 (1332/3021) | 1.26 (1.21-1.31) | 1.24 (1.19-1.29) |
| *Myocardial infarction only b* | 40.1 (425/1061) | 1.27 (1.19-1.36) | 1.24 (1.16-1.32) |
| *Cerebrovascular disease only b* | 52.1 (265/509) | 1.60 (1.47-1.74) | 1.56 (1.44-1.69) |
| *Peripheral arterial diseases only b* | 50.9 (209/411) | 1.49 (1.36-1.63) | 1.45 (1.33-1.59) |
| *Heart failure only b* | 48.4 (89/184) | 1.50 (1.29-1.75) | 1.41 (1.21-1.65) |
| No CVD (reference) | 24.5 (35422/144401) | 1 | 1 |

^1^Adjusted for age and sex. ^2^Further adjusted for remoteness of residence and education.

^a^ Based on self-report and hospital records ^b^Based on hospital records only and participants with CVD subtypes other than the named particular type of CVD subtype were excluded. Effect sizes were estimated using ‘no CVD’ as the reference group.

**(Supplement analysis: Sensitivity analysis-III)**

## **S15 Table.** Retirement*: prevalence and adjusted prevalence ratios according to different CVD-subtype in combination defined based on hospitalisation records

|  | **Retired**  **% [n/N]** | **Prevalence ratio (95% CI)** | |
| --- | --- | --- | --- |
|  |  | **Model^1^** | **Model^2^** |
| IHD and Cerebrovascular disease combined ^a^ | 66.4 (95/143) | 1.81 (1.61-2.03) | 1.73 (1.53-1.95) |
| IHD and HF combined ^a^ | 64.7 (154/238) | 1.72 (1.57-1.89) | 1.65 (1.50-1.81) |
| IHD, HF and PAD combined ^a^ | 75.8 (25/33) | 1.86 (1.53-2.25) | 1.74 (1.44-2.11) |
| Stroke and HF combined ^a^ | 78.1 (25/32) | 2.10 (1.71-2.58) | 1.99 (1.61-2.46) |
| IHD, Cerebrovascular disease and PAD combined ^a^ | 75.0 (18/24) | 1.80 (1.51-2.14) | 1.80 (1.51-2.15) |
|  |  |  |  |
| Main analysis CVD ^b^ | 41.6 (7975/19161) | 1.28 (1.25-1.30) | 1.25 (1.23-1.28) |
| No self-reported or hospital recorded CVD ^b^ (Ref) | 24.5 (35422/144401) | 1 | 1 |

^*^Combination of different subtypes were reported where sufficient numbers were available.

^1^Adjusted for age and sex. ^2^Further adjusted for remoteness of residence and education.

^a^Hospital admission only, ^b^ both self-report and hospital admission, IHD= Ischaemic heart disease, HF= Heart failure, PAD= Peripheral arterial disease

**(Supplement analysis)**

## **S7 Fig.** Retirement due to ill health: prevalence and adjusted prevalence ratios according to CVD subtype among those who have retired and who had not been in paid workforce

|  | **Retired due to ill health**  **% (n/N)** | **Prevalence ratio (95% CI)** | |  |
| --- | --- | --- | --- | --- |
| **Total n/N** | 31.7 (9404/29654) | **Model^1^** | **Model^2^** |  |
| CVD ^a^ | 53.0 (3166/5970) | 1.95 (1.89-2.01) | 1.88 (1.82-1.94) |  |
| *Ischaemic heart disease ^b^* | 62.3 (992/1593) | 2.19 (2.09-2.29) | 2.08 (1.99-2.18) |  |
| *Myocardial infarction ^b^* | 63.8 (247/387) | 2.16 (1.99-2.34) | 2.06 (1.90-2.23) |  |
| *Cerebrovascular disease ^b^* | 72.9 (237/325) | 2.50 (2.33-2.68) | 2.46 (2.29-2.64) |  |
| *Peripheral arterial diseases ^b^* | 73.7 (233/316) | 2.54 (2.36-2.72) | 2.40 (2.23-2.59) |  |
| *Heart failure ^b^* | 81.9 (172/210) | 2.82 (2.62-3.04) | 2.62 (2.43-2.83) |  |
| *Other CVD* ^a^ | 47.0 (1826/3883) | 1.78 (1.71-1.85) | 1.73 (1.66-1.80) |  |
| No CVD (reference) | 26.3 (6238/23684) | 1 | 1 |  |
|  |  |  |  |  |

Model^1^= Adjusted for age and sex, Model^2^ = Adjusted for age-group, sex, remoteness of residence and education attainment. ^a^Based on both self-reported survey and hospital records,  ^b^Based on hospital records only and regardless of other CVD diagnosis. Effect sizes were estimated using ‘no CVD’ as the reference group.

**(Supplement analysis: Sensitivity analysis-I)**

## **S16 Table.** Retirement due to ill health: prevalence and adjusted prevalence ratios according to CVD status where CVD was defined based on hospitalisation records and self-reported survey

|  | Retirement due to ill health **% [n/N]** | **Prevalence ratio (95% CI)** | |
| --- | --- | --- | --- |
|  |  |  |  |
|  |  | **Model^1^** | **Model^2^** |
| Hospital recorded CVD (regardless of self-reported CVD) ^a^ | 59.6 (1758/2952) | 2.11 (2.04-2.19) | 2.02 (1.95-2.10) |
| Self-reported CVD (regardless of hospitalisation recorded CVD) ^b^ | 53.5 (2669/4990) | 1.97 (1.90-2.03) | 1.90 (1.84-1.97) |
|  |  |  |  |
| Hospital recorded CVD only (excluding those with self-reported CVD) ^c^ | 50.7 (497/980) | 1.85 (1.73-1.97) | 1.76 (1.65-1.87) |
| Self-reported CVD only (excluding those with hospitalisation recorded CVD) ^c^ | 46.7 (1408/3018) | 1.78 (1.70-1.86) | 1.73 (1.66-1.81) |
|  |  |  |  |
| Both self-reported and hospitalisation recorded CVD ^c^ | 63.9 (1261/1972) | 2.24 (2.15-2.33) | 2.15 (2.07-2.24) |
|  |  |  |  |
| Main analysis CVD ^c^ | 53.0 (3166/5970) | 1.95 (1.89-2.01) | 1.88 (1.82-1.94) |
| No self-reported or hospital recorded CVD ^c^ (Ref) | 26.3 (6238/23684) | 1 | 1 |

^1^Adjusted for age and sex. ^2^Further adjusted for remoteness of residence and education.

^a^Based on hospital admission only, ^b^ Based on self-report only, ^c^ Based on both self-report and hospital admission

**(Supplement analysis: Sensitivity analysis-II)**

## **S17 Table.** Retirement due to ill health: prevalence and adjusted prevalence ratios according to CVD status excluding those with multiple CVD subtypes

|  | Retirement due to ill health **% [n/N]** | **Prevalence ratio (95% CI)** | |
| --- | --- | --- | --- |
| **Total n/N** | 31.7 (9404/29654) | **Model^1^** | **Model^2^** |
| CVD ^a^ | 53.0 (3166/5970) | 1.95 (1.89-2.01) | 1.88 (1.82-1.94) |
| *Ischaemic heart disease only b* | 57.6 (583/1013) | 2.05 (1.94-2.17) | 1.95 (1.85-2.07) |
| *Myocardial infarction only b* | 60.9 (185/304) | 2.06 (1.88-2.26) | 1.98 (1.80-2.17) |
| *Cerebrovascular disease only b* | 69.4 (152/219) | 2.38 (2.17-2.61) | 2.35 (2.14-2.57) |
| *Peripheral arterial diseases only b* | 68.0 (119/175) | 2.34 (2.11-2.59) | 2.20 (1.98-2.45) |
| *Heart failure only b* | 77.1 (54/70) | 2.71 (2.38-3.08) | 2.51 (2.19-2.88) |
| No CVD (reference) | 26.3 (6238/23684) | 1 | 1 |

^1^Adjusted for age and sex. ^2^Further adjusted for remoteness of residence and education.

^a^ Based on self-report and hospital records ^b^Based on hospital records only and participants with CVD subtypes other than the named particular type of CVD subtype were excluded. Effect sizes were estimated using ‘no CVD’ as the reference group

**(Supplement analysis: Sensitivity analysis-III)**

## **S18 Table.** Retirement due to ill health*: prevalence and adjusted prevalence ratios according to different CVD-subtype in combination defined based on hospitalisation records

|  | Retirement due to ill health **% [n/N]** | **Prevalence ratio (95% CI)** | |
| --- | --- | --- | --- |
|  |  |  |  |
|  |  | **Model^1^** | **Model^2^** |
| IHD and Cerebrovascular disease combined ^a^ | 82.3 (65/79) | 2.73 (2.44-3.05) | 2.68 (2.37-3.03) |
| IHD and HF combined ^a^ | 83.5 (111/133) | 2.88 (2.63-3.15) | 2.67 (2.43-2.94) |
| IHD, HF and PAD combined ^a^ | 95.5 (21/22) | 3.45 (3.06-3.89) | 3.24 (2.81-3.74) |
| Stroke and HF combined ^a^ | 91.3 (21/23) | 3.02 (2.58-3.53) | 2.91 (2.46-3.44) |
| IHD, Cerebrovascular disease and PAD combined ^a^ | 86.7 (13/15) | 3.08 (2.50-3.79) | 3.23 (2.54-4.10) |
|  |  |  |  |
| Main analysis CVD ^b^ | 53.0 (3166/5970) | 1.95 (1.89-2.01) | 1.88 (1.82-1.94) |
| No self-reported or hospital recorded CVD ^b^ (Ref) | 26.3 (6238/23684) | 1 | 1 |

^*^Combination of different subtypes were reported where sufficient numbers were available.

^1^Adjusted for age and sex. ^2^Further adjusted for remoteness of residence and education.

^a^Hospital admission only, ^b^ both self-report and hospital admission, IHD= Ischaemic heart disease, HF= Heart failure, PAD= Peripheral arterial disease

## **S8 Fig.** Relation of sociodemographic factors to non-participation in the workforce among those with and without CVD

|  | **CVD** | | |  | No CVD | | |  |
| --- | --- | --- | --- | --- | --- | --- | --- | --- |
|  | Not in paid workforce | | |  | Not in paid workforce | | | P-interaction |
|  | **% (n)** | ^1^**Prevalence ration (95% CI)** | |  | % (n) | ^1^**Prevalence ration (95% CI)** | |  |
| **Age group (years)** |  |  |  |  |  |  |  |  |
| 45-50 | 21.5 (454) | 1 |  |  | 11.4 (3726) | 1 |  | <0.0001 |
| 50-55 | 25.7 (968) | 1.22 (1.11-1.34) |  |  | 13.7 (5350) | 1.20 (1.15-1.24) |  |  |
| 55-60 | 35.0 (2001) | 1.64 (1.51-1.79) |  |  | 24.0 (9526) | 2.05 (1.98-2.13) |  |  |
| 60-65 | 55.9 (4234) | 2.60 (2.40-2.82) |  |  | 46.9 (15356) | 3.95 (3.82-4.07) |  |  |
| **Sex** |  |  |  |  |  |  |  |  |
| Men | 34.7 (3427) | 1 |  |  | 17.2 (10444) | 1 |  | <0.0001 |
| Women | 45.5 (4230) | 1.34 (1.30-1.39) |  |  | 28.1 (23514) | 1.66 (1.63-1.70) |  |  |
| **Region** |  |  |  |  |  |  |  |  |
| Major cities | 36.7 (3478) | 1 |  |  | 21.8 (16331) | 1 |  | 0.7981 |
| Inner regional | 43.9 (3034) | 1.12 (1.08-1.16) |  |  | 26.0 (13040) | 1.10 (1.08-1.12) |  |  |
| More remote | 43.1 (1021) | 1.05 (0.99-1.10) |  |  | 24.9 (4075) | 1.01 (0.98-1.04) |  |  |
| **Marital status** |  |  |  |  |  |  |  |  |
| Not currently married/defacto | 49.7 (2198) | 1.29 (1.24-1.33) |  |  | 28.0 (8130) | 1.17 (1.15-1.20) |  | <0.0001 |
| Married/defacto | 37.1 (5415) | 1 |  |  | 22.4 (25639) | 1 |  |  |
| **Highest Education** |  |  |  |  |  |  |  |  |
| No school certificate | 63.3 (1556) | 2.55 (2.39-2.71) |  |  | 45.8 (5146) | 2.66 (2.57-2.74) |  | <0.0001 |
| Certificate/diploma/trade | 41.0 (5039) | 1.70 (1.60-1.80) |  |  | 25.0 (22488) | 1.60 (1.56-1.64) |  |  |
| Tertiary | 22.4 (943) | 1 |  |  | 13.9 (5824) | 1 |  |  |
| **Language other than English** |  |  |  |  |  |  |  |  |
| Yes | 41.8 (678) | 1.18 (1.11-1.24) |  |  | 26.0 (3751) | 1.25 (1.22-1.29) |  | 0.1075 |
| No | 39.8 (6979) | 1 |  |  | 23.2 (30207) | 1 |  |  |
| **County of Birth** |  |  |  |  |  |  |  |  |
| Australia/NZ | 39.6 (6035) | 1 |  |  | 23.0 (25858) | 1 |  | 0.0054 |
| Others | 41.2 (1553) | 1.05 (1.01-1.10) |  |  | 25.3 (7838) | 1.10 (1.08-1.12) |  |  |
|  |  |  |  |  |  |  |  |  |
|  |  |  |  |  |  |  |  |  |
|  |  |  | PR 95% CI on log scale |  |  |  | PR 95% CI on log scale |  |
| ^1^Adjusted for age, sex, remoteness of residence and education. | | | |  |  |  |  |  |

## **S9 Fig.** Relation of health-related factors to non-participation in the workforce in those with and without CVD

|  | **CVD** | | |  | No CVD | | |  |
| --- | --- | --- | --- | --- | --- | --- | --- | --- |
|  | Not in paid workforce | | |  | Not in paid workforce | | | P-interaction |
|  | **% (n)** | ^1^**Prevalence ration (95% CI)** | |  | % (n) | ^1^**Prevalence ration (95% CI)** | |  |
| **Alcohol consumption** |  |  |  |  |  |  |  |  |
| No drinkers | 51.0 (3366) | 1.34 (1.30-1.39) |  |  | 31.3 (13050) | 1.37 (1.34-1.40) |  | 0.5358 |
| Moderate drinkers | 33.6 (3102) | 1 |  |  | 19.9 (15673) | 1 |  |  |
| Heavy drinkers | 33.5 (1002) | 1.03 (0.98-1.09) |  |  | 20.2 (4392) | 1.12 (1.09-1.15) |  |  |
| **Smoking status** |  |  |  |  |  |  |  |  |
| Current | 52.6 (1076) | 1.45 (1.38-1.52) |  |  | 30.4 (4059) | 1.39 (1.35-1.43) |  | 0.0039 |
| Past | 40.7 (3192) | 1.09 (1.06-1.13) |  |  | 23.4 (11114) | 1.02 (1.00-1.04) |  |  |
| Never | 36.4 (3348) | 1 |  |  | 22.5 (18650) | 1 |  |  |
| **BMI (**kg/m2) |  |  |  |  |  |  |  |  |
| Underweight (<18) | 56.2 (95) | 1.45 (1.26-1.66) |  |  | 33.3 (456) | 1.39 (1.29-1.49) |  | 0.3785 |
| Normal weight (18‒<25) | 37.2 (1733) | 1 |  |  | 22.4 (11221) | 1 |  |  |
| Overweight Over weight (25‒<30) | 35.8 (2490) | 0.95 (0.91-1.00) |  |  | 21.4 (11155) | 0.96 (0.94-0.98) |  |  |
| Obese ((≥ 30) | 45.6 (2736) | 1.14 (1.10-1.20) |  |  | 27.1 (8449) | 1.11 (1.09-1.14) |  |  |
| **Medical History: Cancer** |  |  |  |  |  |  |  |  |
| No | 38.3 (6216) | 1 |  |  | 22.7 (29227) | 1 |  | 0.2351 |
| Yes | 49.4 (1441) | 1.17 (1.12-1.22) |  |  | 30.7 (4731) | 1.14 (1.11-1.17) |  |  |
| **Medical History: Diabetes** |  |  |  |  |  |  |  |  |
| No | 37.3 (6126) | 1 |  |  | 22.7 (31022) | 1 |  | <0.0001 |
| Yes | 55.6 (1531) | 1.37 (1.32-1.42) |  |  | 37.6 (2936) | 1.38 (1.34-1.42) |  |  |
| **Medical History: Osteoarthritis** |  |  |  |  |  |  |  |  |
| No | 38.7 (7053) | 1 |  |  | 23.0 (32236) | 1 |  | 0.0168 |
| Yes | 65.6 (604) | 1.37 (1.30-1.44) |  |  | 42.8 (1722) | 1.24 (1.20-1.29) |  |  |
|  |  |  |  |  |  |  |  |  |
|  |  |  |  |  |  |  |  |  |
|  |  |  | PR 95% CI on log scale |  |  |  | PR 95% CI on log scale |  |
| ^1^Adjusted for age, sex, remoteness of residence and education. | | | |  |  |  |  |  |

The relation of sociodemographic factors to non-participation in the workforce was similar in those with or without CVD (**S8 Fig.**). The percentage of non-participation in workforce was higher for those aged 55-65 versus 45-55 years, for female versus male, for those living in inner regional and more remote areas versus major cities, for those who are single/widowed/divorced versus married/defacto and for those having less than high school and diploma/trade education versus tertiary education. For both groups (those with or without CVD), non-participation in work was similar for those having language spoken at home other than English versus not having language spoken at home other than English and for those born in countries other than Australia/New Zealand versus Australia/New Zealand, but for those with CVD had higher prevalence of non-participation. Although the percentage of those not in paid workforce of those in different levels of the factors were in the same direction for those living with or without CVD, the PR’s were steeper for all age groups except those in 60-65-year age-group, females, those who live in more remote areas, not currently married, have language spoken at home other than English and born in Australia (**S8 Fig.**).

Among those living with or without CVD, the percentage of non-participation in workforce increased with presence of other comorbid diseases (cancer, diabetes, asthma, osteoarthritis) (**S9 Fig.**). The percentage of those not in paid workforce of those in different levels of the health-related factors were in the same direction for those living with or without CVD. Compared to those living with CVD as a whole, he PR’s were steeper for past and current smokers, those with asthma and all age groups except those in 60-65-year age-group, females, those who live in more remote areas, not currently married, have language spoken at home other than English and born in Australia **S9 Fig.**). Although non-participation in workforce increased for past and current smokers compared with never smoker in those with or without CVD, the gradient is steeper for those living with CVD.
