# Supplementary material for: Cardiovascular disease subtypes, physical disability and workforce participation: A cross-sectional study of 163,562 middle-aged Australians
Source: PLoS One. 2021 Apr 8;16(4):e0249738. doi: 10.1371/journal.pone.0249738 (PMC8031377; doi:10.1371/journal.pone.0249738)
Supplement: Attachment — Submitted filename: ResponseLetter_PONE-D-20-33041_15Jan2021.docx [file pone.0249738.s002.docx]

**RESPONSES TO EDITOR’S AND REVIEWERS’ COMMENTS**

**Editor's comments**

*1. Please ensure that your manuscript meets PLOS ONE's style requirements, including those for file naming. The PLOS ONE style templates can be found at*

[*https://journals.plos.org/plosone/s/file?id=wjVg/PLOSOne_formatting_sample_main_body.pdf*](https://journals.plos.org/plosone/s/file?id=wjVg/PLOSOne_formatting_sample_main_body.pdf) *and*

[*https://journals.plos.org/plosone/s/file?id=ba62/PLOSOne_formatting_sample_title_authors_affiliations.pdf*](https://journals.plos.org/plosone/s/file?id=ba62/PLOSOne_formatting_sample_title_authors_affiliations.pdf)

**We have revised the manuscript to confirm with the requirement of the journal.**

*2. We note that you have indicated that data from this study are available upon request. PLOS only allows data to be available upon request if there are legal or ethical restrictions on sharing data publicly. For information on unacceptable data access restrictions, please see* [*http://journals.plos.org/plosone/s/data-availability#loc-unacceptable-data-access-restrictions*](http://journals.plos.org/plosone/s/data-availability#loc-unacceptable-data-access-restrictions)*.*

*In your revised cover letter, please address the following prompts:*

*a) If there are ethical or legal restrictions on sharing a de-identified data set, please explain them in detail (e.g., data contain potentially identifying or sensitive patient information) and who has imposed them (e.g., an ethics committee). Please also provide contact information for a data access committee, ethics committee, or other institutional body to which data requests may be sent.*

*b) If there are no restrictions, please upload the minimal anonymized data set necessary to replicate your study findings as either Supporting Information files or to a stable, public repository and provide us with the relevant URLs, DOIs, or accession numbers. Please see* [*http://www.bmj.com/content/340/bmj.c181.long*](http://www.bmj.com/content/340/bmj.c181.long) *for guidelines on how to de-identify and prepare clinical data for publication. For a list of acceptable repositories, please see* [*http://journals.plos.org/plosone/s/data-availability#loc-recommended-repositories*](http://journals.plos.org/plosone/s/data-availability#loc-recommended-repositories)*.*

*We will update your Data Availability statement on your behalf to reflect the information you provide.*

**We have added one paragraph in the cover letter to address the issue of data availability, including the restrictions on sharing a de-identified data, and the ways to access the data.**

*3. Thank you for stating the following in your Competing Interests section:*

*'None'*

*a. Please complete your Competing Interests on the online submission form to state any Competing Interests. If you have no competing interests, please state "The authors have declared that no competing interests exist.", as detailed online in our guide for authors at* [*http://journals.plos.org/plosone/s/submit-now*](about:blank)

*b. This information should be included in your cover letter; we will change the online submission form on your behalf.*

*Please know it is PLOS ONE policy for corresponding authors to declare, on behalf of all authors, all potential competing interests for the purposes of transparency. PLOS defines a competing interest as anything that interferes with, or could reasonably be perceived as interfering with, the full and objective presentation, peer review, editorial decision-making, or publication of research or non-research articles submitted to one of the journals. Competing interests can be financial or non-financial, professional, or personal. Competing interests can arise in relationship to an organization or another person. Please follow this link to our website for more details on competing interests:* [*http://journals.plos.org/plosone/s/competing-interests*](about:blank)

**In the cover letter above, we have stated about the grants received by authors, and declared that the authors have no other competing interests.**

***Reviewer #1:***

*The authors examined the relationship of cardiovascular disease to workforce participation in older working-age people. For this purpose they used questionnaire data from a very large population based study assessed between 2006 and 2009 in New South Wales (Australia) and linked it to hospitalisation data. The study size of more than 160,000 subjects is enormous. The study is comprehensibly described and written in good English. Nevertheless, I do have some comments/questions:*

**We thank the reviewer for recognising the importance of this work.**

***Major***

*1. How do the authors explain the observation that older people (60-64 years) have lower PRs than people aged 50-55 years (see Fig 3)?*

**The higher PR in the younger age group is not surprising.  The PR is the prevalence (proportion) in the exposed divided by the prevalence in the unexposed (baseline) group. Thus, all else being equal, as the prevalence in the baseline group increases, the PR will decrease. Because the prevalence of (adverse) outcomes commonly increase with age, it is very common to see PRs decrease with age, as is the case here.**

**Crude prevalence (proportion) of workforce non-participation was higher in 60-64 year olds compared to 50-54 year olds, irrespective of CVD status; see the following extract from Fig 3.**

| **Age-group** | **Proportions (%) not in work** | | **PR (95% CI)** |
| --- | --- | --- | --- |
|  | **CVD group** | **No CVD group** |  |
| **50-54 year** | **25.7** | **13.7** | **1.75 (1.65-1.84)** |
| **60-64 year** | **56.0** | **47.0** | **1.21 (1.19-1.24)** |

**The following statement has been included in line 253 to 254 in the manuscript:**

**Although the crude prevalence of workforce non-participation was higher in older compared to younger age groups (irrespective of CVD status), the relation of CVD and workforce non-participation became weaker with increasing age.**

*2. The data analysed are relatively old. How do the authors assess the transferability of the results to the present day? Could the results be influenced by the financial crisis of 2007-2008?*

**Population distribution and cardiovascular disease profile in Australia have not drastically changed since these data were collected. Notably, the effect of the financial crisis of 2007-2008 on Australia has been considerably less than in many other countries (ABS, 2010). Regardless, the relative estimates (PRs) comparing those CVD vs without may well be transferable (Rothman et al., 2013**)**. This is particularly so if the financial crisis or other factors have not affected workforce participation disproportionately based on CVD status, but this is unlikely.**

**Reference:**

**ABS (Australian Bureau of Statistics), The Global Financial Crisis and its impact on Australia, Year Book Australia 2009–10, Number 91, ABS Canberra, ABS Catalogue No. 1301.0, Chapter 27 — Financial system, 2010, p. 687-88.**

**Rothman KJ, Gallacher JE, Hatch EE. Why representativeness should be avoided. Int J Epidemiol. 2013 Aug;42(4):1012-4. doi: 10.1093/ije/dys223. PMID: 24062287**

*3. I stumble upon the number of subjects included. Perhaps the relevant section could be made clearer:
• In the abstract the subjects with CVD (19,137) and without CVD (144,294) do not add up to 163,562.*

**Thank you for identifying this. The final number of eligible participants was 163,562; this is the number of participants included in the descriptive tables. As outcome-specific exclusions for missing or invalid data were applied for each outcome, the number of participants contributing to regression models were slightly different: 163,431 for workforce participation, 114,064 for paid hours of work and 11,961 for retirement due to ill health. However, there was no missing value for retirement. We have modified the abstract, methods and study flow chart (Fig S1) to make this clearer.**

45 and Up Study participants n=267,153

Excluded: invalid data on age or date of recruitment, n=454 those with linkage errors, n=195 age>=65 at baseline, n=102,942

Working age (45-<65 years) at baseline, n=163,562 (CVD group, n=19161 No CVD group, n=144,401)

With valid workforce participation status, n=163,431 (CVD group, n=19137 No CVD group, n=144294)

With valid data on retirement due to ill health, n=29,654 (CVD group, n=5,970 No CVD group, n=23,684)

With valid data on paid hours of work, n=114,064 (CVD group, n=10,506 no CVD group, n=103,558)

***S1 Fig. Flowchart for selection of participants***

*• How large was the data set finally evaluated?*

**We have modified the abstract, methods and study flow chart (Fig S1) to make this clearer.**

*4. The references should be revised. For example, in some references the PID is given in others it is missing.*

**We have checked all references again, aiming to provide PIDs. However, not all references had a PID, hence it was not possible to provide a PID for all references. In the first draft, we used Endnote as reference manager but in this revised version the references were edited manually after unlinking those from the main body of the article.**

**Minor**
*• Should ‘Language other than English (LOTE)’ describe the mother tongue? (Line 144) Please specify.*
**The questionnaire asked ‘Do you speak a language other than English at home’ followed by two options to choose from (yes and no). To further clarify, the wording has been modified both in the text and in the table to ‘language spoken at home other than English (yes/no)’.**

*• Why were Australia and New Zealand combined when considering the place of birth (Line 144)?*
**New Zealand citizens do not need a visa to live or work in Australia and have access to most of the same entitlements, including health and welfare, and opportunities as Australian citizens. Hence, we grouped Australian and New Zealand citizens as one category and classified participants from other countries as others.**

*• Please explain the basis for the categorisation of alcohol consumption. How were the cut-offs chosen? How was an alcoholic drink defined? (Lines 146-148)*

**This variable was categorised with cut points (0, 1-14 and ≥15) aimed to align with the Australian alcohol consumption guidelines to minimise the risk of long-term harm. At the time of data collection, the guideline for low-risk alcohol consumption in Australia was daily consumption of 20 gram of alcohol and the standard drink was defined as 10 gram of alcohol. This meant 14 standard drinks per week was considered low-risk alcohol drink for both men and women in Australia at the time of data collection. The following text with reference is added in the ‘Other variables of interest section’ of main text of the manuscript.**

**The cut-points of alcohol consumption broadly reflect Australian guidelines on low-risk consumption [33].**

**References**

**33. NHMRC (National Health and Medical Research Council). Australian Guidelines To Reduce Health Risks From Drinking Alcohol (NHMRC, Canberra, 2009).**

*• How were the cut-offs chosen for physical functioning limitations? (Lines 153-156)*
**We have added a reference for the choice of cut points for physical functioning limitations. The manuscript now reads:**

**Physical functioning limitations scores ranged from 0 to 100, with higher scores representing fewer limitations, and were grouped into four categories: no limitation (score of 100); minor limitation (score 90–99); moderate limitation (60–89); and severe limitation (score 0–59) [35]**

**35. Stewart A, Kamberg C. Measuring functioning and well-being: the Medical Outcomes Study Approach.Durham, North Carolina: Duke University Press; 1992**

**Further details are provided in Supplementary Table S6.**

*• The information on the exclusion of persons without data regarding workforce participation should be given in the method section. Please specify how many subjects were excluded. (Lines 173/174)*
**We have updated the methods section of the manuscript to clarify the definition. The following paragraph has been added to the end of ‘Study population and data sources’.**

**The participants with invalid data on age or date of recruitment (n=454), those with linkage errors (n=195) and those aged 65 years or over (n=102,942) were excluded. There were 163,562 participants who were of working age (45-<65 years old) with known CVD status at baseline (70 458 men, 93 104 female) (details in supplementary file: S1-S4 Figs, S1-S3 Tables).**

**At the end of ‘Outcome: Workforce participation’ section, the following statement regarding the number of participants excluded is added.**

**Outcome-specific exclusions for missing or invalid data were applied for each outcome. Of the 163,562 participants included in the study, 131 had missing data on workforce participation status (Supplementary Fig1). Of the 121,816 participants in the work force, 7752 had missing/invalid data on the number of paid hours per week. Of the 41,615 participants not in the work force, 11,961 had invalid data on retirement due to ill health.**

*• Table 1: What does „/“ stand for in the 3rd line (Percentage (%))?*

**This section in the table is revised to ‘/163562’ in both columns.**

*• Inaccuracies in Table 2: It should be „[inter quartile range]“ and „To care for family“ should be shown left centred.*

**We have updated Table 2.**

*• Fig 1: Why do you mention SEM but do not show SEM?*

**The value is very small and thus might not be always visible. A new note ‘‘, and SEM might not be always visible because of small values’’ is embedded in Figure 2. The mean and standard error of mean (SEM) of paid work hours per week according to CVD, age-group are presented for ‘men and women combined’, men and women. These values were used to generate the bar diagram presented in the paper. The mean values were rounded while printing those on top of the figure but while making the bar height, the values upto 5 decimal points as follows were used.**

**Table: Mean and standard error of mean for paid work hours per week according stratified by age-group, CVD status and sex.**

|  |  |  | **Men and women combined** | |  | **Men** | |  | **Women** | |
| --- | --- | --- | --- | --- | --- | --- | --- | --- | --- | --- |
| **Age-groups** | **CVD status** |  | **Mean*** | **SEM**** |  | **Mean*** | **SEM**** |  | **Mean*** | **SEM**** |
| **45-<50yr** | **No CVD** |  | **32.98442** | **0.10004** |  | **41.03347** | **0.147298** |  | **27.81902** | **0.120497** |
| **45-<50yr** | **CVD** |  | **28.57292** | **0.444737** |  | **35.28957** | **0.707296** |  | **23.89315** | **0.531286** |
| **50-<55yr** | **No CVD** |  | **32.07892** | **0.095206** |  | **39.41718** | **0.139583** |  | **26.99338** | **0.117408** |
| **50-<55yr** | **CVD** |  | **27.33017** | **0.341942** |  | **33.20252** | **0.514454** |  | **22.14744** | **0.422722** |
| **55-<60yr** | **No CVD** |  | **26.53238** | **0.104639** |  | **34.01773** | **0.158582** |  | **20.88362** | **0.126173** |
| **55-<60yr** | **CVD** |  | **22.53104** | **0.289037** |  | **27.70834** | **0.41672** |  | **16.66617** | **0.362824** |
| **60-<65yr** | **No CVD** |  | **15.4578** | **0.111741** |  | **21.28552** | **0.185435** |  | **10.71942** | **0.123857** |
| **60-<65yr** | **CVD** |  | **12.41692** | **0.217996** |  | **16.13304** | **0.325377** |  | **7.795259** | **0.25165** |

*** mean of paid work hours per week, **Standard error of mean of paid work hours per week, CVD= Cardiovascular disease, yr= years old**

***Reviewer #2****:*

*Overall, the manuscript provides valuable information on CVD and workforce participation, based on a large sample size.*

**We thank the reviewer for recognising the importance of this work.**

*Please consider following comments:*

*1. The response rate should already be mentioned in the methods, briefly, and - if applicable - further aspects with regard to sample composition (weighting?, exclusions due to implausible questionnaires?).*

**A detailed description of the 45 and Up study is available as part of a published protocol (Banks et al,. International journal of epidemiology. 2008;37(5):941-7. doi: 10.1093/ije/dym184) so we did not extensively describe it in this paper. However, we have added in further details on the response rate, weighting and exclusion criteria to the revised manuscript. The newly added texts in the ‘Study population and data sources’ section are as follows:**

**Individuals joined the study by completing a postal questionnaire (available at https://www.saxinstitute.org.au/our-work/45-up-study/questionnaires/) and providing consent for follow-up through linkage to a range of routinely-collected data. The majority of participants joined in 2008, with the majority completing the baseline questionnaire in February 2008. Persons aged 80 years and over and those living in rural and remote areas were oversampled by a factor of two. The response rate to mailed invitations was estimated to be 18%, with the final sample representing around 10% of the NSW population aged 45 years and older.**

**The participants with invalid data on age or date of recruitment (n=454), those with linkage errors (n=195) and those aged 65 years or over (n=102,942) were excluded. There were 163,562 participants who were of working age (45-<65 years old) with known CVD status at baseline (70 458 men, 93 104 female) (details in supplementary file: S1-S4 Figs, S1-S3 Tables).**

*2. Outcome measurement:
Full retirement definition was based on giving reasons for retirement, why not just use the information on current work status (“Completely retired/pensioner”)?*

**Most participants chose more than one option from the multiple-choice questions that asked about current work status and many were found to be in apparent conflict regarding retirement status. On the other hand, defining retirement status from the questionnaire that asked about the cause of retirement was straight forward. Note we have used the term ‘retired’, and have not differentiated between ‘full-retirement’ or ‘partial retirement’.**

*Current work status was indicated by multiple choice, did multiple choices occur and how was this handled then? Participation in work was also defined by paid work hours including 0 hours?*

**Yes, there were participants choosing more than one option from the multiple-choice questions. Logical checks were done using responses to questions on current work status and ‘paid work hours per week’. We have made this clearer in the revised manuscript (first paragraph of the section ‘Outcomes: Workforce participation’ in page 6). The steps of defining workforce participation might explain further how paid work hours per week contributed to the definition of workforce participation, and how inconsistencies between paid work status and paid work hour per week were resolved. The following text is added in the supplementary section in S4 Table.**

1. **Participants were considered to be in paid work if:**
   1.1. **Number of hours of paid work hours is valid (0 to <100) OR**
   1.2. **Reported being in full time paid work, in part time paid work, self-employed or partially retired.**
2. **Participants were considered to be not in paid work if:**
   2.1. **Number of hours of paid work hours per week is zero (0) or missing (but not invalid) AND**
   2.2. **Not reported being in fulltime paid work, part time paid work, self-employed or partially retired**
3. **People who are not paid for work automatically received zero (0) for paid work hour per week**
4. **For those who are in “paid” category and entered 0 as paid work hour per week, their paid work status was accepted, and weekly paid work hours were invalidated**
5. **For those who are in “not paid” category and entered valid paid work hours per week, their paid work status was changed, and weekly paid work hours were accepted when the weekly paid work hours were larger than 0.**

*3. I could not find the number of excluded participants with missing data on workforce participation (either text or flow chart).*

**The proportion is mentioned at the footnote of Table 2. The exact numbers are now mentioned in response to first reviewer’s query number 3 as above and also, in the supplementary file (S1-S4 Figs and S1-S3 Tables).**

*4. There seems to be some truncated text in the two tables.*

**We have updated the both tables.**

*5. The overlap of workforce participation/retirement should not be considered in analysis retirement (line 116). Do the precentages in line 210/11 refer to this, though? Then it could also be included in table 2.*

**This has been updated and the exact numbers are now mentioned in the text with relevant wordings. It was explained in query 3 of first reviewer, and further details are provided in the supplementary file (S1-S4 Figs and S1-S3 Tables).**

*6. Line 281: The "and" in the middle should be "or"?*

**We have updated this sentence and now in line 295 in the clean version.**

*7. As the authors correctly state in the methods, causal relationships cannot be assessed in this study, though implicitly assumed at least in conclusions. Lines 289 ff seem to discuss possible causal relationships, but the paragraph needs more comprehensible and clear formulation. This should also include physical disablity.*

**This paragraph has been updated and includes the issue of physical disability as a possible underlying mechanism which is further explained in the paragraph that follows it. The wording is as follows:**

**Another underlying reason for higher workforce non-participation in people with CVD could be physical disability since physical functioning has been shown to decline following incident CVD [41] and this study has indicated that those with higher physical functioning limitations had the highest probability of being out of paid-work.**

**Reference:**

**41.	Kucharska-Newton A, Griswold M, Yao ZH, Foraker R, Rose K, Rosamond W, et al. Cardiovascular Disease and Patterns of Change in Functional Status Over 15 Years: Findings From the Atherosclerosis Risk in Communities (ARIC) Study. J Am Heart Assoc. 2017;6(3):e004144. pmid: 28249844.**

*8. I am not familiar with "PRs, which are based on internal comparisons" (line 324), what does this mean/what would be the opposite? Despite the reference44, I doubt that it is correct to negate any selection bias in the study. As education was used in the analysis (and is usually associated with CVD), how comparable was it to the general population?*

**The ratio of prevalences of two groups is called prevalence ratio (PR), and it is analogous to the risk ratio (RR) in cohort studies. Although the absolute proportions estimated in this study will likely not reflect the absolute numbers in the target population, it is reasonable to assume that the relative comparison between groups (in this case the PRs of CVD compared to non-CVD) will be similar in the target population if confounding is minimised (see also response to Reviewer 1, point 2).** **We have acknowledged the possibility of selection bias but also emphasised the strength of findings from internal comparison in this investigation. The wording of the sentence is now updated as follows:**

**Hence, while our absolute estimates of CVD prevalence and workforce participation may not be generalisable, PRs, which are based on internal comparisons, are still likely to be generalisable [47].**

**47.	Mealing NM, Banks E, Jorm LR, Steel DG, Clements MS, Rogers KD. Investigation of relative risk estimates from studies of the same population with contrasting response rates and designs. BMC Med Res Methodol. 2010;10:26. pmid: 20356408**

**We sought to look for other comparable studies but could not find any that investigated the association of CVD, workforce participation and education among study participants similar to ours.**

*9. As results for physical disablity are a "key finding", they should also be included in the conclusions.*
**The issue of physical disability was previously mentioned at the end of the sentence (in line 337 of the old version and line 409 of the new version). Now in this version we have deleted the end section of that sentence and added a new sentence stating it as a key finding in the conclusion (in line 362-363 of new clean version), which is as follows.**

**Physical functioning limitations was a key factor in non-participation in the paid workforce both for those with and without CVD.**

*10. "The results of this investigation have significant policy implications for healthy and successful ageing for those living with CVD." (line 339ff) I cannot follow this: What are the implications for health for people with CVD here?*

**The following text is added at the end of the discussion section to clarify the implications (last section of the final paragraph of the discussion section).**

**Our results show the majority of people with CVD were still in the paid workforce but people who experienced a CVD event, especially those with physical disability, were more vulnerable to not being in the workforce compared to their counterparts. Given the importance of paid work for individual wellbeing and the overall economy [15], policies aimed at helping people with CVD, particularly those with accompanying physical disability, to remain or re-enter the workforce would be of benefit to both individuals and society.**

**Reference:**

**15. Schofield D, Shrestha R, Percival R, Passey M, Callander E, Kelly S. The personal and national costs of CVD: impacts on income, taxes, government support payments and GDP due to lost labour force participation. Int J Cardiol. 2013;166(1):68-71. pmid: 22018513.**

**Editor's comments**

*PLOS authors have the option to publish the peer review history of their article (*[*what does this mean?*](https://journals.plos.org/plosone/s/editorial-and-peer-review-process#loc-peer-review-history)*). If published, this will include your full peer review and any attached files.
If you choose “no”, your identity will remain anonymous but your review may still be made public.*

**We agree to publish the full peer review history.**

With best wishes,

Yours sincerely,

**Muhammad Shahdaat Bin Sayeed**, on behalf of the authors

ORCID ID: [0000-0003-0027-9614](https://orcid.org/0000-0003-0027-9614)
